# The complete mitochondrial genome of *Neurigona zhejiangensis* (Diptera: Dolichopodidae)

**DOI:** 10.1080/23802359.2020.1851155

**Published:** 2021-01-12

**Authors:** Juan Wang, Yutong Ji, Lisheng Zhang, Mengqing Wang

**Affiliations:** Institute of Plant Protection, Chinese Academy of Agricultural Sciences, Beijing, China

**Keywords:** Mitochondrial genome, Dolichopodidae, phylogenetics

## Abstract

The long-legged fly *Neurigona zhejiangensis* belongs to the subfamily Neurigoninae of Dolichopodidae. The mitogenome (GenBank accession number: MT921956) of *N. zhejiangensis* was sequenced, the first representative complete mitogenome from this subfamily. The complete mitogenome is 17,141 bp, includes 13 protein-coding genes, 2 rRNAs, and 22 transfer RNAs. All genes have the same location and coding strand as in other published species of Dolichopodidae. Nucleotide composition is biased toward A and T, which together made up 77.3% of the entire genome. Bayesian inference strongly supported the monophyly of Empidoidea, Empididae and Dolichopodidae, with the phylogenetic relationships within Empidoidea: (Dolichopodinae + Neurigoninae) + ((Empidinae + (Trichopezinae + Oreogetoninae)) + Ocydromiinae).

## Introduction

*Neurigona* Rondani, 1856 belongs to the subfamily Neurigoninae. It is a large genus of Dolichopodidae with 158 known species (Yang et al. 2006, 2011; Wang et al. 2007, 2010, 2020). The long-legged flies or Dolichopodidae are one of the largest groups in Brachycera. They are predacious, including some important predators of bark beetles (Scolytidae) and mosquitoes (Culicidae), except for the larvae of *Thrypticus* which are phytophagous (Yang et al. 2006).

The specimens of *N. zhejiangensis* used for this study were collected in Suiyang County of Guizhou Province by Mengqing Wang and identified by Mengqing Wang. Specimens were deposited in the Natural Enemy Insects Museum (Accession Number: NI2017-1) of the Institute of Plant Protection, Chinese Academy of Agricultural Sciences (IPPCAAS) (Room 311, Plant Protection Building). Total genomic DNA was extracted from a whole body (except head) specimen using the QIAamp DNA Blood Mini Kit (Qiagen, Germany) and stored at −20 °C until needed. 1 μg of genomic DNA was used to generate libraries with an average insert size of 350 bp, which were sequenced using the Illumina NovaSeq 6000 platform (Berry Genomics, Beijing, China) with 150 bp paired-end reads on one sample per flow-cell lane. A total of 21,354,824 raw paired reads were generated. The quality of all sequences was checked using FastQC (http://www.bioinformatics.babraham.ac.uk/projects/fastqc). Then, the clean paired reads were generated with the software AdapterRemoval version 2 (Schubert et al. 2016). There reads were assembled into the contigs via NOVOPlasty (Dierckxsens et al. 2017). The assembled genome was annotated using the MITOS webserver with the invertebrate genetic code (Bernt et al. 2013). The complete mitogenome of *N. zhejiangensis* is 17,141 bp (GenBank accession number: MT921956) and encoded 13 PCGs, 22 tRNA genes, and 2 rRNA genes (Qilemoge et al. 2019; Yang et al. 2019; Qilemoge et al. 2020). All genes have the same location and coding strand as in other published species of Dolichopodidae. Nucleotide composition was biased toward A and T, with 77.3% A + T content (A = 41.4%, T = 35.9%, C = 14.0%, G = 8.7%). The A + T content of PCGs, tRNAs, and rRNAs was 73.4, 76.1, and 80.0%, respectively. Six PCGs (*NAD2*, *COI*, *ATP8*, *NAD3*, *NAD5*, and *NAD6*) are initiated by ATT codons, and six PCGs (*COII*, *COIII*, *ATP6*, *NAD4*, *NAD4L*, and *CYTB*) are initiated by ATG codons, with *NAD1* initiated by ATA. All PCGs used the typical termination codons TAA (10 of 13) while *NAD3*, *CYTB*, and *NAD1* used TAG.

**Figure 1.**
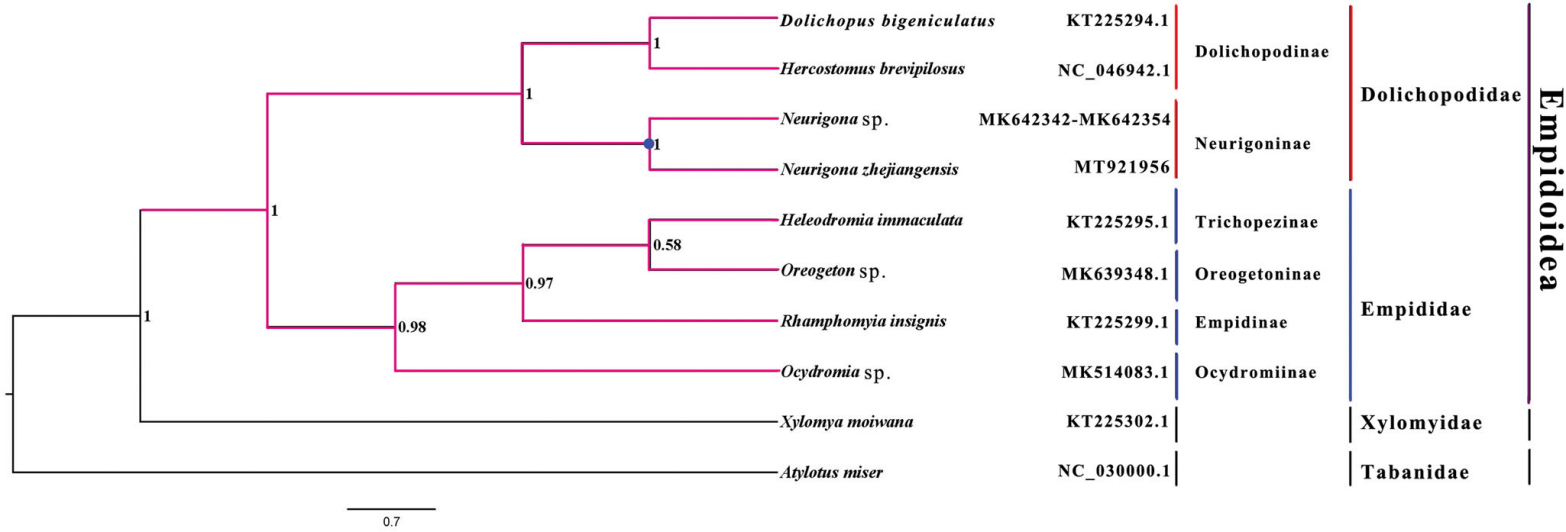
Bayesian phylogenetic tree of 10 Diptera species. The posterior probabilities are labeled at each node. Genbank accession numbers of all sequences used in the phylogenetic tree have been included in figure and corresponding to the names of all species.

Phylogenetic analysis was performed based on the nucleotide sequences of 13 PCGs from 10 Diptera species. Sequences were aligned using MAFFT v7.313 (Katoh and Standley 2013) and the Bayesian Inference (BI) tree was constructed with PhyloBayes 4.1 (Lartillot et al. 2009), which was run for 10,000,000 generations and sampled from every 1000 generations. The CAT + GTR model selected by ModelFinder (Kalyaanamoorthy et al. 2017) was applied in the BI analysis. The phylogenetic result strongly supported the monophyly of Empidoidea, Dolichopodidae, and Empididae. Monophyletic Dolichopodinae and Neurigoninae were grouped as a monophyletic Dolichopodidae, which was a sister group to a monophyletic Empididae that consists of Empidinae, Trichopezinae, Oreogetoninae, and Ocydromiinae in this study (Figure 1). Monophyly of Empididae and Dolichopodidae is consistent with previous phylogenetic results (Wang et al. 2016). The complete mitogenome of *N. zhejiangensis* contributes to further phylogenetic analysis and taxonomic studies of the Empidoidea.

## Data Availability

The data that support the findings of this study are openly available in [NCBI] at [https://www.ncbi.nlm.nih.gov/], reference number [MT921956].
